# Effect of general anesthesia on postoperative pulmonary embolism

**DOI:** 10.1080/07853890.2025.2530228

**Published:** 2025-07-10

**Authors:** Junnan Xu, Xinyuan Yu, Yilong Shi, Fengyu Chen, Liang Wang, Jie Weng, Zhiyi Wang, Yingbin Deng

**Affiliations:** ^a^ Department of Emergency Medicine, The Second Affiliated Hospital and Yuying Children’s Hospital of Wenzhou Medical University, Wenzhou, China; ^b^ Department of General Practice, Lu’an Hospital of Anhui Medical University, Lu’an, China; ^c^ Department of Public Health, Marshall University, Huntington, WV, USA; ^d^ Department of General Practice, The Second Affiliated Hospital and Yuying Children’s Hospital of Wenzhou Medical University, Wenzhou, China; ^e^ Wenzhou Key Laboratory of Precision General Practice and Health Management, Wenzhou, China; ^f^ Department of Organ Fibrosis Research Center, South Zhejiang Institute of Radiation Medicine and Nuclear Technology, Wenzhou, China

**Keywords:** Anesthesia type, the duration of anesthesia time, pulmonary embolism

## Abstract

**Background:**

The influence of anesthesia type and duration on the occurrence of pulmonary embolism (PE) after surgery remains controversial. This study investigates the association between anesthesia type and duration with postoperative PE.

**Methods:**

A retrospective cohort of adult patients undergoing surgery from May 2020 to August 2024 at large-scale general hospitals was analyzed. Multivariable logistic regression models were employed to adjust for potential confounders, and sensitivity analyses (using overlap weighting and array approach) were performed to validate the findings.

**Results:**

A total of 178,052 patients were included in the analysis, of whom 91 developed PE after surgery. The median duration of general anesthesia (GA) was 1.72 h, with an interquartile range (IQR) of 1.17–2.52 h. The median duration of regional anesthesia was 1.54 h, with an IQR of 1.20–2.03 h. Anesthesia type and the duration of regional anesthesia were not associated with PE occurrence (adjusted odds ratio [aOR] [95% confidence interval, CI], 1.148 [0.671–2.098], *p* = 0.631), (aOR [95% CI], 1.117 [0.498–1.557], *p* = 0.738). The rates of PE consistently increased with GA prolongation (aOR [95% CI], 1.308 [1.176–1.432], *p* < 0.001). Compared with GA durations < 3 h, prolonged anesthesia was significantly associated with increased PE incidence (aOR [95% CI], 4.398 [2.585–7.565], *p* < 0.001). These findings were also confirmed by sensitivity analyses.

**Conclusions:**

Our study demonstrates that prolonged GA, particularly > 3 h, significantly increases the risk of PE.

## Introduction

Venous thromboembolism (VTE), which includes deep vein thrombosis (DVT) and pulmonary embolism (PE), contributes considerably to perioperative morbidity and mortality [1]. Particularly, PE is a serious complication due to its sudden and often fatal presentation [2]. Since anesthesia is critical in surgery, identifying modifiable PE risk factors requires careful examination of its role [3]. The debate regarding the influence of general anesthesia (GA) versus regional anesthesia (RA) on the risk of PE, as well as the impact of anesthesia duration, has profound implications for patient safety and surgical outcomes [4–6].

Recent studies have reported conflicting findings about the association between anesthesia type and the risk of postoperative PE. Yap et al. [7] and Saied et al. [8] reported that GA does not significantly increase the risk of postoperative PE. However, Morgan et al. [9] observed that patients who underwent hip fracture surgery under RA had a higher propensity to develop DVT and PE compared to those who received GA. A meta-analysis conducted by Chen et al. [10] concluded that GA did not significantly influence the incidence of PE within the context of hip fracture surgery. The discordance in findings regarding the impact of anesthesia type on PE underscores the necessity for further investigation. Additionally, anesthesia duration may influence PE occurrence. Furthermore, increased surgical time may increase patient immobility, thereby increasing the risk of thrombosis. Despite the biological plausibility of this relationship, current empirical evidence has produced inconsistent results. Kim et al. [3] indicated that longer operative time is indeed associated with higher PE rates, while Masuda et al. [11] found no notable association after adjusting for confounding variables.

Variability across studies likely stems from differences in: study designs, patient groups, surgery types, and multiple confounding factors affecting anesthesia duration and PE risk [7,9]. Additionally, most prior studies used univariate methods and ignored how anesthesia type-duration interactions might alter postoperative PE risk [10,11]. Therefore, the present study aims to investigate the effects of anesthesia type and duration on the risk of postoperative PE. By leveraging a large database, we aim to provide more definitive answers to these questions and contribute to the ongoing discourse on how anesthesia practice can be optimized to reduce the risk of postoperative PE.

## Methods

### Study design and setting

**Figure 1.**
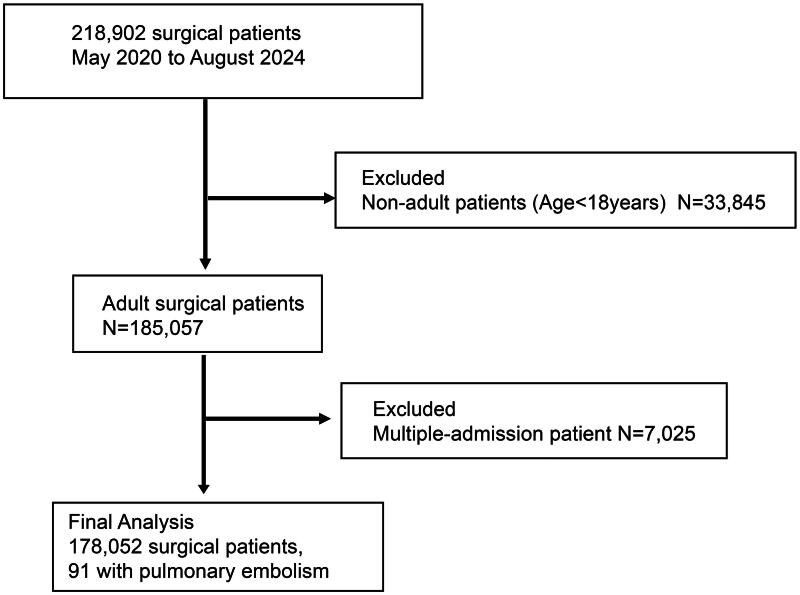
Subject inclusion/exclusion flow diagram.

This retrospective cohort study extracted data from the integrated healthcare system of the Second Affiliated Hospital and Yuying Children’s Hospital of Wenzhou Medical University. The study period spanned from May 2020 to August 2024, encompassing patients aged ≥18 years who underwent inpatient surgery (Figure 1). Eligibility criteria were based on the ICD-10-CM classification system for identifying surgical types. Only the first admission record of patients with multiple inpatient surgery records within the 4-year study frame was chosen for analysis. The study protocol was aligned with the STROBE guidelines. The Ethics Review Committee of the Second Affiliated Hospital of Wenzhou Medical University approved the study protocol, with a waiver for patient informed consent due to its retrospective nature (2024-K-148-01).

### Covariates

Demographic characteristics comprised age, sex, and body mass index (BMI). Surgical factors included the urgency of surgery (elective vs. emergency), anesthesia type (general vs. regional), recovery time, surgical sequence, surgery site, surgical classification, the American Society of Anesthesiologists (ASA) physical status grade, age-adjusted Charlson Comorbidity Index (aCCI) and comorbidities assessed through the 5-year look-back period, urine volumes, bleeding volume, effusion, assistant anesthesiologist, and instrument nurse. The study also recorded anesthesia and surgery duration.

### Outcome measures and exposure

The primary outcome measure was the development of postoperative PE diagnosed by pulmonologists or intensivists based on the established clinical criteria and confirmed by imaging studies such as computed tomography angiography or ventilation-perfusion scans [12]. The primary exposure variable was anesthesia type, which included parameters such as total anesthesia time and specific intervals (e.g. ≤3 h vs. ≥3 h).

### Missing data

In the context of this retrospective study, we anticipated potential gaps in data, particularly regarding comorbidities, laboratory values, and procedural details. To address missing data, we employed multiple imputation using chained equations, which is a flexible approach to handle missing values by modeling each variable as a function of others.

### Statistical analysis

Data without normal distribution are presented as median (interquartile range, IQR), and categorical data are expressed as percentages. Initial univariate associations for categorical and continuous predictors were assessed using chi-square (χ^2^) and Mann-Whitney U tests, respectively. Multivariable logistic regression models were employed to evaluate the independent association between anesthesia type, anesthesia duration, and the risk of postoperative PE, adjusting for potential confounders such as age, sex, BMI, anesthesia type, ASA, aCCI, urine volume, bleeding volume, and surgical classification. The adjusted changes in the expected PE incidence were assessed using the average marginal effects approach. We calculated the adjusted risk within varying groups of anesthesia duration to enhance the reliability of our findings.

Subgroup analyses were conducted to assess the interaction between anesthesia duration and the risk of postoperative PE within different surgery types (e.g. orthopedic). Sensitivity analyses were performed by reevaluating the primary associations in various patient subgroups, including expanding the age criterion to ≥65 years, to ensure the robustness of our findings. Furthermore, multivariable logistic regression models were used to explore these interactions. Second, we employed overlap weighting (OW) [13,14] as an alternative method to mitigate indication bias when examining the relationship between anesthesia duration and PE. We utilized the array approach [15] sensitivity analysis, incorporating resampling, to elucidate potential residual confounding attributable to unmeasured variables.

We conducted a preplanned exploratory analysis to assess whether the relationship between PE and anesthesia duration was influenced by patient characteristics, surgery site, and surgery urgency. This involved incorporating an interaction term into the multivariable logistic regression model, reflecting the characteristics of interest, which included patient age, sex, surgery site, and aCCI.

All statistical analyses were conducted using R software version 4.4.0 (R Foundation for Statistical Computing, Vienna, Austria). A *P-*value <0.05 indicated statistical significance for all analyses, employing a two-tailed test.

## Results

**Figure 2.**
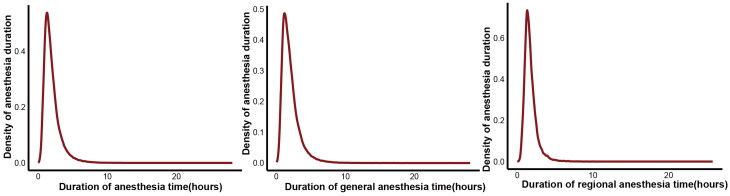
Density plot of observed duration of anesthesia time.

From May 2020 to August 2024, 218,902 surgical cases were extracted from the hospital system. After excluding non-adult patients and those with multiple admissions, 178,052 patients were included in the analysis. Among them, 137,863 patients received GA, 40,189 patients received RA, 4,805 required intensive care unit stay postoperatively, and 91 patients experienced PE (Table 1, Figure 1). The median GA duration was 1.72 h, with an IQR of 1.17–2.52 h. For RA, the median duration was 1.54 h, with an IQR of 1.20–2.03 h (Figure 2). The incidence of PE did not significantly differ between patients receiving GA and those receiving RA (Table 1).

**Table 1.** Characteristics of surgical patients.

Characteristic	Total *N* = 178052	General anesthesia *N* = 137863	Regional anesthesia *N* = 40189	*P* Value
Demographics				
Male sex, n (%)	82394 (46.3%)	62843 (45.6%)	19551 (48.6%)	<0.001
Age, Median (IQR, years)	48[36;57]	49 [39;58]	42 [32;55]	<0.001
BMI, Median (IQR)	24.0 [21.9;26.0]	24.0 [21.6;25.7]	24.2 [22.6;27.0]	<0.001
Surgical information				
Emergency, n (%)	15375 (8.64%)	8643 (6.27%)	6732 (16.8%)	<0.001
Surgical duration, Median (IQR), h	1.74 [1.25;2.48]	1.80 [1.25;2.60]	1.61 [1.26;2.10]	<0.001
Duration of anesthesia time, Median (IQR), h	1.67 [1.17;2.39]	1.72 [1.17;2.52]	1.54 [1.20;2.03]	<0.001
Recovery time, Median (IQR), h	0.60 [0.46;0.78]	0.62 [0.50;0.82]	NA	NA
Surgical sequence, Median (IQR)	3 [2;5]	3 [2;5]	3 [2;5]	<0.001
Site of surgery				<0.001
Orthopedic surgery, n (%)	93189 (52.3%)	66890 (48.5%)	26299 (65.4%)	
General abdominal surgery, n (%)	20334 (11.4%)	20093 (14.6%)	241 (0.60%)	
Urological surgery, n (%)	10617 (5.96%)	10442 (7.57%)	175 (0.44%)	
Cardiac and vascular surgery, n (%)	3409 (1.91%)	3096 (2.25%)	313 (0.78%)	
Other surgical procedures, n (%)	50503 (28.4%)	37342 (27.1%)	13161 (32.7%)	
Surgical Classification, n (%)				<0.001
I	19439 (10.9%)	15076 (10.9%)	4363 (10.9%)	
II	54121 (30.4%)	37702 (27.3%)	16419 (40.9%)	
III	82550 (46.4%)	65074 (47.2%)	17476 (43.5%)	
IV	21942 (12.3%)	20011 (14.5%)	1931 (4.80%)	
ASA, n (%)				0.818
I	63688 (35.8%)	49761 (36.1%)	13927 (34.7%)	
II	106285 (59.7%)	81164 (58.9%)	25121 (62.5%)	
III	6170 (3.47%)	5084 (3.69%)	1086 (2.70%)	
IV	1537 (0.86%)	1486 (1.08%)	51 (0.13%)	
V	367 (0.21%)	364 (0.26%)	3 (0.01%)	
aCCI, Median (IQR)	1.00 [0.00;1.00]	1.00 [0.00;2.00]	0.00 [0.00;1.00]	<0.001
Volumes of urine, Median (IQR), ml	0.00 [0.00;150]	0.00 [0.00;200]	0.00 [0.00;100]	<0.001
Bleeding volume, Median (IQR), ml	10.0 [5.00;50.0]	10.0 [5.00;50.0]	20.0 [10.0;300]	<0.001
Effusion, Median (IQR), ml	0.00 [0.00;0.00]	0.00 [0.00;0.00]	0.00 [0.00;0.00]	<0.001
Assistant anesthesiologist, n (%)	11469 (6.44%)	10524 (7.63%)	945 (2.35%)	<0.001
Instrument nurse, n (%)	48793 (27.4%)	39554 (28.7%)	9239 (23.0%)	<0.001
Outcome				
Postoperative admitted to ICU, n (%)	4805 (2.70%)	4400 (3.19%)	405 (1.01%)	<0.001
Pulmonary embolism, n (%)	91 (0.05%)	76 (0.06%)	15 (0.04%)	0.206

BMI: Body Mass Index. ASA: American Society of Anesthesiologists Physical Status Classification System. aCCI: Age-adjusted Charlson Comorbidity Index. ICU: Intensive Care Unit.

**Figure 3.**
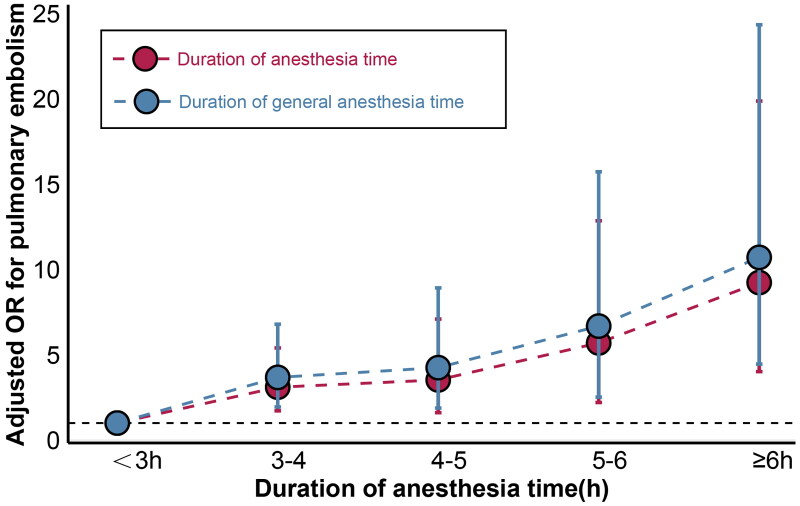
The association of duration of anesthesia time and PE development.

After adjustment, anesthesia type (GA or RA) was not associated with PE occurrence (adjusted odds ratio [aOR] [95% confidence interval, CI], 1.148 [0.671–2.098], *p* = 0.631). RA duration is not associated with PE occurrence (aOR [95% CI], 1.117 [0.498–1.557], *p* = 0.738). PE rates consistently increased with GA duration (aOR [95% CI], 1.308 [1.176–1.432], *p* < 0.001) (Table 2). The probability of PE occurrence increased by 0.015% (95% CI: 0.009%–0.021%) for each additional hour of GA (Table S1). Compared with GA lasting <3 h, prolonged GA was significantly associated with an increased incidence of PE (aOR [95% CI], 4.398 [2.585–7.565], *p* < 0.001) (Table 2). When considering anesthesia duration at each fixed interval independently (relative to 3-h anesthesia), the risk increase was linear for PE (Figure 3). When GA lasted <3 h, the incidence of PE did not increase with GA prolongation (Table S3). The absolute proportion of PE closely approximated its adjusted proportion (Table S2).

**Table 2.** Adjusted association of duration of anesthesia time and pulmonary embolism following surgery.

	Total *N* = 178052		General anesthesia *N* = 137863		Regional anesthesia *N* = 40189	
Variable	OR(95%CI)	*P*	OR(95%CI)	*P*	OR(95%CI)	*P*
Duration of anesthesia time, h	1.297(1.172–1.408)	<0.001	1.308(1.176–1.432)	<0.001	1.117(0.498–1.557)	0.738
Duration of anesthesia time > 1h	0.667(0.359–1.358)	0.228	0.687(0.347–1.519)	0.312	0.896(0.228–5.951)	0.890
Duration of anesthesia time > 2h	1.966(1.213–3.249)	0.007	2.166(1.246–3.900)	0.008	1.912(0.599–5.702)	0.251
Duration of anesthesia time > 3h	3.734(2.309–6.033)	<0.001	4.398(2.585–7.565)	<0.001	1.498(0.081–7.970)	0.703
Duration of anesthesia time > 4h	3.462(1.995–5.871)	<0.001	3.623(2.039–6.328)	<0.001	NA	NA
Duration of anesthesia time > 5h	4.043(2.097–7.459)	<0.001	3.980(2.026–7.507)	<0.001	NA	NA
Duration of anesthesia time > 6h	3.912(1.757–8.068)	<0.001	3.719(1.638–7.829)	0.001	NA	NA
Duration of anesthesia time interval						
<2h	Reference					
2–3h	0.999(0.505–1.891)	0.997	0.895(0.382–1.950)	0.788	1.848(0.534–5.750)	0.301
3–4h	3.098(1.643–5.725)	<0.001	3.528(1.765–7.024)	<0.001	2.387(0.126–13.755)	0.422
4–5h	3.526(1.549–7.457)	0.002	4.071(1.719–9.140)	0.001	NA	NA
5–6h	5.690(2.136–13.453)	<0.001	6.398(2.315–15.999)	<0.001	NA	NA
≥6h	9.223(3.837–20.938)	<0.001	10.224(4.017–24.894)	<0.001	NA	NA
<3h	Reference					
3–4h	3.100(1.720–5.400)	<0.001	3.675(1.943–6.790)	<0.001	1.893(0.102–10.001)	0.638

**Figure 4.**
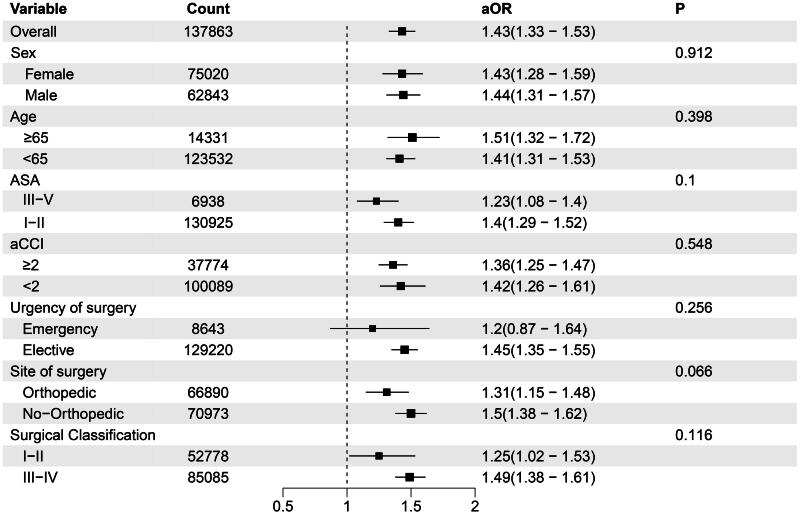
Variation in the adjusted duration of anesthesia time and PE development according to patient and clinical factors.

The sensitivity analysis showed similar study outcomes in the subgroups of orthopedic surgery, elective surgery, and patients aged ≥65 years (Table S3). OW using patients’ propensity for GA duration within 3 h–matched pairs yielded well-balanced groups (Figure S1). Sensitivity analyses revealed that the risks of PE associated with GA lasting >3 h were consistent with those obtained from the primary analysis utilizing multivariable logistic regression (Table S4). The array-based sensitivity analyses demonstrated that if the prevalence of unmeasured confounding factors in the prolonged anesthesia group exceeds that in the short anesthesia group by >40%, the relative risk for PE must be at least 8.0 to change the significance of our findings (Table S5 and Table S6). In 97.9% of 1000 bootstrap replications, the incidence of PE and extended GA showed a significant correlation (Figure S2). We observed no statistical evidence that the relationship between GA duration and PE incidence was influenced by factors such as patient age, sex, urgency of surgery, or surgical classification (Figure 4).

## Discussion

Further elucidation of the association between anesthesia type, particularly GA, and the risk of postoperative PE can significantly influence clinical decision-making and enhance perioperative management strategies [7,8]. This insight may facilitate the development of tailored prophylactic measures, enabling anesthesiologists, surgeons, and other healthcare providers to make informed decisions regarding the most appropriate anesthetic technique to balance surgical outcomes with patient safety. Our comprehensive analysis, leveraging a large-scale database, addresses a critical gap in the literature by examining the impact of anesthesia duration and type on PE incidence across various surgical specialties and settings.

The findings of this study revealed that the risk of postoperative PE was significantly increased and associated with GA compared to RA. Notably, this elevated risk appears to manifest only when GA exceeds 3 h. In contrast, procedures performed under RA did not demonstrate a similar increase in postoperative PE risk. These results underscore the importance of considering the type and duration of anesthesia when evaluating the potential for thromboembolic complications in surgical patients. Additionally, we observed a similar trend in GA duration with PE occurrence after adjusting for confounding factors in other surgery types.

The biological mechanisms underlying this association are complex [16]. GA induces systemic physiological changes [17], including alterations in hemodynamics and respiratory function, which can predispose patients to venous stasis and subsequent thrombus formation. Furthermore, the immobility associated with longer surgical procedures exacerbates these risks because prolonged periods of inactivity can lead to blood pooling in the lower extremities [18–20]. This observation aligns with the well-established concept of ‘Virchow’s triad’ [21] stating that venous stasis, hypercoagulability, and endothelial injury are critical factors in the development of venous thromboembolism [20]. It is noteworthy that the requirement for endotracheal intubation and controlled mechanical ventilation during GA introduces specific physiological changes that may further increase the risk of PE, particularly with prolonged duration. Positive pressure ventilation elevates intrathoracic pressure, impedes venous return, and reduces cardiac output, thereby exacerbating venous stasis [22]. Additionally, mechanical ventilation can induce ventilation-perfusion mismatch and hypoxemia, which is a known stimulant for coagulation and endothelial dysfunction [23,24]. While our retrospective data limit definitive attribution, this mechanistic pathway plausibly contributes to the observed association between prolonged GA and PE.

In a study analogous to ours, Sager et al. [25] identified that an elevated risk of VTE was associated with GA and surgeries exceeding 80 min during rotator cuff repair; however, they did not explore the specific implications for PE. Saied et al. [8] found that GA also increased the risk of DVT but reported no evidence of any effect on postoperative PE in their study utilizing the US National Surgical Quality Improvement database. This absence of effect may be attributed to their inclusion criterion, which restricted surgery duration to ≤4 h, potentially mitigating the observed impact of anesthesia type on PE. Previous literature generally suggested that GA does not influence the occurrence of postoperative PE [7,10,11]. Nonetheless, postoperative PE is a relatively low-probability event that poses challenges for a study due to the insufficient number of positive cases needed to achieve statistically significant result [26,27]. Interestingly, Morgan et al. [9] observed that spinal anesthesia, in contrast to GA, was associated with a significantly higher risk of DVT and PE following hip fracture surgery. Patients predisposed to VTE due to underlying risk factors or limited mobility may preferentially receive spinal anesthesia, potentially increasing the observed incidence of DVT and PE in their cohort. Notably, a few studies considered the impact of anesthesia duration on postoperative PE, which may help elucidate the lack of association between GA and PE observed in the aforementioned studies. Although Phillips et al. [28] explored the effects of prolonged GA duration on VTE, the small sample size of their study—comprising only one case of PE and one case of DVT among the outcome events—limits the ability to accurately assess the impact of anesthesia duration on PE. In a retrospective cohort study involving over 1.4 million surgical patients from >130 hospitals, Kim et al. [3] identified a direct association between prolonged surgical duration and an increased risk for DVT and PE. However, the specific impact of anesthesia duration on the risk of PE was not explicitly determined by calculating a z-score to standardize anesthesia duration across procedures. Our study clarifies the influence of anesthesia duration on the risk of postoperative PE, which is important for guiding surgical management and preventive strategies against PE.

Clinically, the association between anesthesia duration and postoperative PE occurrence highlights the importance of anesthesia timing in evaluating the risk of PE following surgery. Currently, two primary risk stratification tools for VTE are the Caprini [29] and Rogers [30] scores. However, the Rogers score does not consider anesthesia duration, while the Caprini score only distinguishes between procedures classified as ‘major surgery’ based on a duration threshold of 45 min, which may not adequately reflect the influence of anesthesia on the VTE risk. Additionally, the Wells [31] and revised Geneva score [32], which are frequently used to predict PE, do not factor anesthesia duration in their assessments. Despite the value of these scoring systems for their predictive capabilities, they may overlook a crucial variable by failing to consider the potential effects of prolonged anesthesia on the risk of developing PE.

Our findings further indicate that prolonged GA (exceeding 3 h) may be an independent and modifiable risk factor for PE. Incorporating this variable into existing risk assessment tools, such as the Caprini score, could enhance the accuracy of risk stratification and may aid in better identifying high-risk patients who require intensified preventive measures, such as extended pharmacological anticoagulation or mechanical compression. Given the substantial number of annual surgeries performed, even a minor increase in the risk of PE associated with prolonged anesthesia can cause a considerable burden [11]. These data prompt us to reconsider the combination of procedures or the extension of longer operations [25]. They underscore the necessity for advancements reducing GA duration or, when feasible, the implementation of alternative GA methods, particularly in surgical environments susceptible to thromboembolic complications.

The ongoing healthcare reforms in China, similar to the International Patient Protection and Affordable Care Act, are exerting increasing pressure on medical professionals to mitigate postoperative complications, which elevate readmission rates and healthcare expenditures. PE, a critical and potentially fatal complication, underscores the importance of risk quantification in optimizing the quality and efficiency of patient care. This focus on PE underscores the necessity for precise risk assessment to guide clinical decision-making and develop targeted prophylactic strategies, ultimately aiming to alleviate the healthcare burden associated with this serious condition. This emphasis on quantification is particularly highlighted by key regulatory bodies and organizations in China that have established mandates for PE risk reduction and the implementation of prophylactic strategies. Our findings not only contribute to the postoperative risk assessment but also serve as a crucial benchmark for PE rates, facilitating the evaluation of future initiatives aimed at reducing PE risks and their associated healthcare burdens.

The present study endeavored to elucidate the relationship between anesthesia type, duration, and the risk of postoperative PE. Despite the robustness of our findings, several limitations must be acknowledged. First, our study is based on a retrospective cohort design, which, while providing valuable insights, might cause inherent biases that may influence the causality of the observed associations. Key parameters such as mechanical ventilation settings and postoperative mobilization records were unavailable, limiting mechanistic interpretation of the association between anesthesia duration and PE risk. Additionally, our reliance on the database from a single healthcare system may limit the generalizability of our results to other populations or healthcare settings. Our study specifically focused on PE, with all cases being diagnosed based on the established clinical criteria and confirmed by definitive imaging studies such as computed tomography angiography or ventilation-perfusion scans, thereby ensuring clarity and precision of our outcomes. However, this approach may not capture all instances of subclinical PE [33], potentially influencing the reported incidence. Another limitation is the potential for unmeasured confounders that could affect anesthesia duration and PE risk. Although we adjusted for various demographic, surgical, and comorbid factors, other variables, such as intraoperative prophylactic anticoagulation strategies (e.g. dosage and timing of heparin use), hemodynamic instability events (e.g. duration of hypotension), and inherited thrombophilia (e.g. coagulation factor gene mutations), that were not accounted for might have existed in our analysis. Meanwhile, the relatively low absolute number of PE events (*n* = 91) may reduce the statistical power to detect weak associations, particularly in subgroup analyses. Nevertheless, prolonged GA (>3 h) still demonstrated a strong effect size (aOR95% CI = 4.40(2.59–7.57)), and the sensitivity analysis yielded robust results, suggesting that its clinical significance is not compromised by this limitation.

Despite these limitations, our study presents several crucial advantages. The comprehensive nature of our analysis, encompassing diverse surgical procedures and patient characteristics, facilitates a more nuanced understanding of the factors contributing to postoperative PE. Our rigorous statistical approach, including multiple imputation for handling missing data, multivariable logistic regression, OW, and array approach to control for confounding factors, enhances the reliability of our results. Focusing on anesthesia type and duration in relation to PE constitutes a novel contribution to the literature, providing insights that support clinical decision-making and perioperative management strategies. Additionally, our study addresses a critical gap in the literature and offers more definitive answers regarding the impact of anesthesia practices on postoperative PE risk by leveraging a large-scale database. Furthermore, our findings have practical implications for developing prophylactic measures and risk assessment tools. The association between anesthesia duration and the occurrence of postoperative PE emphasizes the necessity for considering anesthesia duration in the assessment of PE risk. This insight can guide anesthesiologists, surgeons, and other healthcare providers in selecting the most appropriate anesthesia to balance optimal surgical outcomes with patient safety.

## Conclusion

In conclusion, we demonstrated that prolonged general anesthesia, particularly when exceeding 3 h, is considerably associated with an increased risk of PE. Our findings highlight the importance of considering anesthesia duration when assessing the risk of PE, which could lead to more tailored and effective perioperative management.

## Supplementary Material

Supplemental Material

Figure S2.jpg

supplement materials.docx

Figure S1.jpg

## Data Availability

The data supporting the findings of this study are available from the corresponding author upon reasonable request.
