# Supplementary material for: Effect of general anesthesia on postoperative pulmonary embolism
Source: Ann Med. 2025 Jul 10;57(1):2530228. doi: 10.1080/07853890.2025.2530228 (PMC12247096; doi:10.1080/07853890.2025.2530228)
Supplement: Supplemental Material [file IANN_A_2530228_SM1820.docx]

Figure S1 Comparison of standardized mean differences for the unweighted sample after OW.

Figure S2 Density Plot and Caterpillar Plot for association of GA lasting >3 h and PE development.
