# Supplementary figures and images for: Effect of general anesthesia on postoperative pulmonary embolism
Source: Ann Med. 2025 Jul 10;57(1):2530228. doi: 10.1080/07853890.2025.2530228 (PMC12247096; doi:10.1080/07853890.2025.2530228)

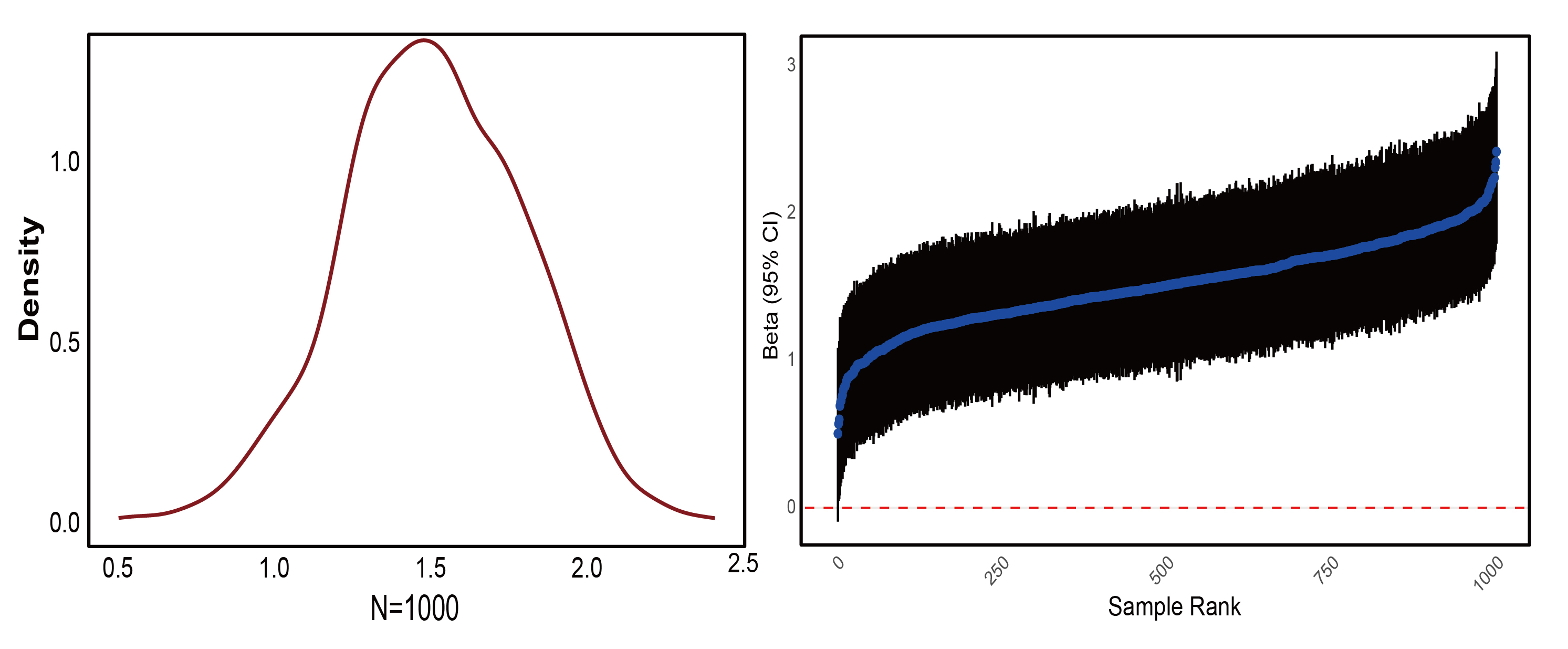

Supplement: Figure S2.jpg [file IANN_A_2530228_SM8032.jpg]

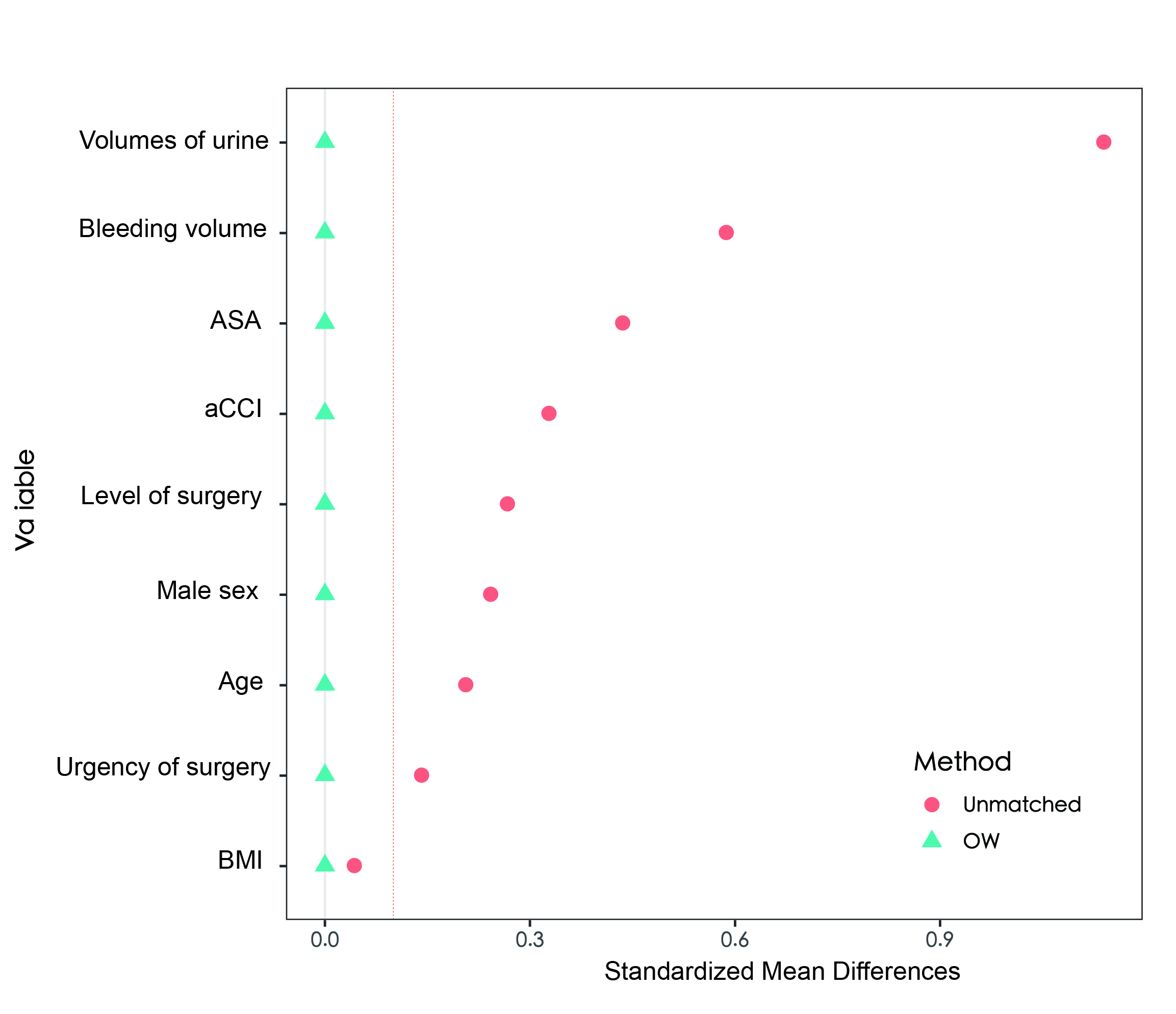

Supplement: Figure S1.jpg [file IANN_A_2530228_SM8030.jpg]
