# Supplementary material for: Effect of general anesthesia on postoperative pulmonary embolism
Source: Ann Med. 2025 Jul 10;57(1):2530228. doi: 10.1080/07853890.2025.2530228 (PMC12247096; doi:10.1080/07853890.2025.2530228)
Supplement: supplement materials.docx [file IANN_A_2530228_SM8031.docx]

**Supplementary material**

**Variable definition**

"Surgical duration" is defined as the time interval from the patient's entry into the operating room to their exit from it.

"Duration of anesthesia time" is defined as the time span commencing from the initiation of anesthesia to its termination

"Recovery time" is defined as the interval from the cessation of anesthesia and the commencement of recovery until the completion of the recovery process, during which the patient regains consciousness.

"Surgical sequence" refers to the order in which a particular surgery is scheduled within the operating room.

“Surgical Classification” in accordance with the 'Management Measures for Surgical Classification in Medical Institutions' issued by the National Health Commission of the People's Republic of China in 2022.

**Sensitivity analyses**

The analysis of the relationship between anesthesia duration and the incidence of PE is confounded by other risk factors, which arises here because drivers of anesthesia duration (disease severity) also influences the occurrence of PE^[1]^. In addition to the primary analysis employing multivariable logistic regression to control for confounding, we employed two sensitivity analyses using alternative statistical methods — Overlap Weighting (OW)^[2, 3]^ and array approach^[4]^ — to control for confounding by indication.

For the overlap weighting (OW) analysis, we initially constructed a propensity score to evaluate the effect of duration of GA >3 hours. The covariates incorporated in the propensity score model were consistent with those utilized in the primary analysis for adjustment. This propensity score was subsequently applied for OW in a logistic regression model assessing the relationship between prolonged GA duration and the occurrence of PE. A graphical assessment (Supplemental Figure 1) indicated satisfactory predictor balance post-OW, with the weighted outcomes closely mirroring the original outcomes (Supplemental Table 4).

The Array Approach involves quantifying the strength of unmeasured confounders and their imbalance between exposure groups to assess the impact of these factors on relative risk estimates. We evaluated the impact of unmeasured confounding factors on our study results by adjusting for the association between the unmeasured confounder and the exposure, the prevalence of the unmeasured confounder in the exposed group , the prevalence of the unmeasured confounder in the unexposed group, and the relative risk after accounting for the unmeasured confounder. The study results indicate that if the prevalence of unmeasured confounding factors in the prolonged anesthesia group exceeds that in the short anesthesia group by more than 40%, the relative risk for pulmonary embolism must be at least 8.0 to change the significance of our findings. At an even smaller imbalance in prevalence (i.e. 30%), not even an RRcd of 10 would change the significance of our finding(Table S5, Table S6).

Table S1 Adjusted association of duration of anesthesia time and pulmonary embolism following surgery.

| Variable | Total  N= 178052 | | General anesthesia  N=137863 | | Regional anesthesia  N= 40189 | |
| --- | --- | --- | --- | --- | --- | --- |
|  | Adjust the difference in pulmonary embolism incidence | *P* | Adjust the difference in pulmonary embolism incidence | *P* | Adjust the difference in pulmonary embolism incidence | *P* |
| Duration of anesthesia time, h | 0.013% (0.008%- 0.018%) | <0.001 | 0.015% (0.009% - 0.021%) | <0.001 | 0.004% (-0.020%-0.028%) | 0.739 |
| Duration of anesthesia time>1h | -0.024% (-0.070%- 0.022%) | 0.303 | -0.024% (-0.078% - 0.030%) | 0.383 | -0.004% (-0.066%-0.058%) | 0.894 |
| Duration of anesthesia time>2h | 0.032% (0.010%-0.055%) | 0.005 | 0.038% (0.012% -0/064%) | 0.004 | 0.027% (-0.026%-0.080%) | 0.315 |
| Duration of anesthesia time>3h | 0.084% (0.044% - 0.125%) | <0.001 | 0.095% (0.051% -0.140%) | <0.001 | 0.018% (-0.091%-0.127%) | 0.748 |
| Duration of anesthesia time>4h | 0.096% (0.033% - 0.158%) | 0.003 | 0.102% (0.036%-0.169%) | 0.002 | NA | NA |
| Duration of anesthesia time>5h | 0.128% (0.030% - 0.226%) | 0.010 | 0.130% (0.029% -0.232%) | 0.012 | NA | NA |
| Duration of anesthesia time>6h | 0.131% (0.057%- 0.257%) | 0.041 | 0.129% (0.014% - 0.257%) | 0.048 | NA | NA |
| Duration of anesthesia time interval |  |  |  |  |  |  |
| ＜2h | Reference |  |  |  |  |  |
| 2-3h | -0.00004% (-0.020% - 0.020%) | 0.997 | -0.0003% (-0.024%-0.018%) | 0.785 | 0.025% (-0.030%-0.081%) | 0.370 |
| 3-4h | 0.063% (0.018% - 0.108%) | 0.007 | 0.071% (0.021%-0.121%) | 0.005 | 0.041% (-0.102%-0.184%) | 0.571 |
| 4-5h | 0.076% (0.005% -0.146%) | 0.035 | 0.085% (0.0097%-0.163%) | 0.027 | NA | NA |
| 5-6h | 0.140% (0.004% -0.276%) | 0.043 | 0.151% (0.007%-0.295%) | 0.039 | NA | NA |
| ≥6h | 0.244% (0.051%-0.437%) | 0.013 | 0.257% (0.052%-0.462%) | 0.014 | NA | NA |
| ＜3h | Reference |  |  |  |  |  |
| 3-4h | 0.063% (0.019% - 0.108%) | 0.006 | 0.072% (0.023%-0.122%) | 0.004 | 0.032% (-0.104%-0.168%) | 0.645 |
| 4-5h | 0.076% (0.060% - 0.145%) | 0.033 | 0.088% (0.011%-0.164%) | 0.024 | NA | NA |
| 5-6h | 0.140% (0.005% - 0.275%) | 0.042 | 0.153% (0.009%-0.297%) | 0.037 | NA | NA |
| ≥6h | 0.244% (0.053% - 0.436%) | 0.013 | 0.261% (0.055%-0.466%) | 0.013 | NA | NA |

Table S2. Sensitivity analyses of general anesthesia with pulmonary embolism.

| Anesthesia time | Event of pulmonary embolism | Absolute Proportion | Adjusted Proportion | 95%CI | | Adjusted Risk Difference | 95%CI | |
| --- | --- | --- | --- | --- | --- | --- | --- | --- |
| ≤1h | 9 | 0.038% | 0.038% | 0.020% | 0.073% | Reference | | |
| >1h | 67 | 0.059% | 0.059% | 0.046% | 0.074% | 0.020% | 0.008% | 0.036% |
| ≤2h | 19 | 0.023% | 0.023% | 0.015% | 0.036% | Reference | | |
| >2h | 57 | 0.105% | 0.105% | 0.081% | 0.136% | 0.082% | 0.058% | 0.113% |
| ≤3h | 28 | 0.024% | 0.024% | 0.017% | 0.035% | Reference | | |
| >3h | 48 | 0.212% | 0.212% | 0.160% | 0.280% | 0.187% | 0.135% | 0.256% |
| ≤4h | 46 | 0.036% | 0.036% | 0.027% | 0.048% | Reference | | |
| >4h | 30 | 0.303% | 0.303% | 0.213% | 0.433% | 0.267% | 0.177% | 0.397% |
| ≤5h | 56 | 0.042% | 0.042% | 0.032% | 0.055% | Reference | | |
| >5h | 20 | 0.431% | 0.431% | 0.279% | 0.664% | 0.389% | 0.237% | 0.622% |
| ≤6h | 63 | 0.046% | 0.046% | 0.036% | 0.059% | Reference | | |
| >6h | 13 | 0.558% | 0.558% | 0.326% | 0.953% | 0.512% | 0.280% | 0.906% |
| ＜3h | 28 | 0.024% | 0.024% | 0.017% | 0.035% | Reference | | |
| 3-4h | 18 | 0.139% | 0.139% | 0.088% | 0.220% | 0.115% | 0.064% | 0.196% |
| 4-5h | 10 | 0.189% | 0.189% | 0.103% | 0.348% | 0.165% | 0.079% | 0.324% |
| 5-6h | 7 | 0.300% | 0.300% | 0.145% | 0.618% | 0.276% | 0.121% | 0.593% |
| ≥6h | 13 | 0.556% | 0.556% | 0.325% | 0.949% | 0.531% | 0.301% | 0.924% |

Table S3. Sensitivity analyses of the adjusted association of anesthesia time and pulmonary embolism following surgery.

| Sensitivity analysis | Total | | General anesthesia | | Regional anesthesia | |
| --- | --- | --- | --- | --- | --- | --- |
|  | OR（95%CI） | *P* | OR（95%CI） | *P* | OR（95%CI） | *P* |
| Duration of anesthesia time≤1h | 13.877(0.196 - 3145.847) | 0.280 | 20.681(0.149 - 13387.978) | 0.287 | 31.992(0.005 - 113870645.486) | 0.513 |
| Duration of anesthesia time≤2h | 0.561(0.202 - 1.561) | 0.266 | 0.714(0.212 - 2.411) | 0.586 | 0.321(0.05 - 2.132) | 0.230 |
| Duration of anesthesia time≤3h | 0.730(0.414 - 1.266) | 0.268 | 0.653(0.334 - 1.246) | 0.202 | 1.197(0.416 - 3.293) | 0.731 |
| Duration of anesthesia time≤6h | 1.516(1.232 - 1.851) | <0.001 | 1.585(1.267 - 1.971) | <0.001 | 1.162(0.504 - 2.268) | 0.696 |
| Elective surgery | 1.327(1.204 - 1.442) | <0.001 | 1.343(1.210 - 1.472) | <0.001 | 1.015(0.396 - 1.730) | 0.972 |
| Age ≥65years | 1.382(1.116 - 1.676) | 0.002 | 1.418(1.134 - 1.736) | 0.001 | 0.946(0.252 - 2.535) | 0.927 |
| Orthopedic surgery | 1.281(1.019 - 1.491) | 0.006 | 1.263(1.001 - 1.483) | 0.016 | 1.268(0.424 - 1.843) | 0.469 |

Table S4. Sensitivity analyses employing alternative analysis methods to measure pulmonary embolism associated with anesthesia time.

| Analysis method | OR for pulmonary embolism when general anesthesia is >3 hours. (95% CI) | *P* |
| --- | --- | --- |
| Multivariable logistic regression | 4.398(2.585 - 7.565) | <0.001 |
| Propensity-based overlap weighting | 1.377(1.148-1.598) | <0.001 |

Table S5 Array Approach Sensitivity Analysis for Unmeasured Confounder That Increases PE Rate, and Is More Prevalent in the Exposed (Longest Procedures).

The observed relative risk (ARR; approximated by the OR) was set as the lower confidence interval of the pooled odds ratio for the duration of general anesthesia time >3h and PE rate (OR = 4.398; 95% CI = **2.585** - 7.565).

The association between the unmeasured confounder and PE rate (RRCD) was varied from 1 to 10.

The prevalence of this confounder was varied from 10% to 50%.

Abbreviations:

ARR = Observed relative risk

RR_CD_ = Association between the unmeasured confounder and PE

P_C0_= Prevalence of the unmeasured confounder in the exposed (duration of general anesthesia time>3h)

P_C1_= Prevalence of the unmeasured confounder in the unexposed (reference, duration of general anesthesia time ≤3h)

RR= Relative risk after adjusting for the unmeasured confounder

$$RR=\frac{\mathrm{ARR}}{\left[ \frac{P_{C1}\left( \mathrm{RR}_{\mathrm{CD}}-1 \right)+1}{P_{C0}\left( \mathrm{RR}_{\mathrm{CD}}-1 \right)+1} \right]}$$

Bolded scenarios would reduce the lower limit of the 95% confidence interval for the pooled odds ratio below 1.00.

| ARR (95%CI) | RR_CD_ | P_C0_ | P_C1_ | RR (95%CI) |  |  |  |  |  |  |  |  |
| --- | --- | --- | --- | --- | --- | --- | --- | --- | --- | --- | --- | --- |
| 4.398(2.585 - 7.565) | 1 | 0.1 | 0.1 | 4.398(2.585-7.565) |  |  |  |  |  |  |  |  |
| 4.398(2.585 - 7.565) | 2 | 0.1 | 0.1 | 4.398(2.585-7.565) |  |  |  |  |  |  |  |  |
| 4.398(2.585 - 7.565) | 3 | 0.1 | 0.1 | 4.398(2.585-7.565) |  |  |  |  |  |  |  |  |
| 4.398(2.585 - 7.565) | 4 | 0.1 | 0.1 | 4.398(2.585-7.565) |  |  |  |  |  |  |  |  |
| 4.398(2.585 - 7.565) | 5 | 0.1 | 0.1 | 4.398(2.585-7.565) |  |  |  |  |  |  |  |  |
| 4.398(2.585 - 7.565) | 6 | 0.1 | 0.1 | 4.398(2.585-7.565) |  |  |  |  |  |  |  |  |
| 4.398(2.585 - 7.565) | 7 | 0.1 | 0.1 | 4.398(2.585-7.565) |  |  |  |  |  |  |  |  |
| 4.398(2.585 - 7.565) | 8 | 0.1 | 0.1 | 4.398(2.585-7.565) |  |  |  |  |  |  |  |  |
| 4.398(2.585 - 7.565) | 9 | 0.1 | 0.1 | 4.398(2.585-7.565) |  |  |  |  |  |  |  |  |
| 4.398(2.585 - 7.565) | 10 | 0.1 | 0.1 | 4.398(2.585-7.565) | P_C1_ | RR (95%CI) |  |  |  |  |  |  |
| 4.398(2.585 - 7.565) | 1 | 0.2 | 0.1 | 4.398(2.585-7.565) | 0.2 | 4.398(2.585-7.565) |  |  |  |  |  |  |
| 4.398(2.585 - 7.565) | 2 | 0.2 | 0.1 | 4.032(2.370-6.935) | 0.2 | 4.398(2.585-7.565) |  |  |  |  |  |  |
| 4.398(2.585 - 7.565) | 3 | 0.2 | 0.1 | 3.770(2.216-6.484) | 0.2 | 4.398(2.585-7.565) |  |  |  |  |  |  |
| 4.398(2.585 - 7.565) | 4 | 0.2 | 0.1 | 3.573(2.100-6.147) | 0.2 | 4.398(2.585-7.565) |  |  |  |  |  |  |
| 4.398(2.585 - 7.565) | 5 | 0.2 | 0.1 | 3.421(2.011-5.884) | 0.2 | 4.398(2.585-7.565) |  |  |  |  |  |  |
| 4.398(2.585 - 7.565) | 6 | 0.2 | 0.1 | 3.299(1.939-5.674) | 0.2 | 4.398(2.585-7.565) |  |  |  |  |  |  |
| 4.398(2.585 - 7.565) | 7 | 0.2 | 0.1 | 3.199(1.880-5.502) | 0.2 | 4.398(2.585-7.565) |  |  |  |  |  |  |
| 4.398(2.585 - 7.565) | 8 | 0.2 | 0.1 | 3.115(1.831-5.359) | 0.2 | 4.398(2.585-7.565) |  |  |  |  |  |  |
| 4.398(2.585 - 7.565) | 9 | 0.2 | 0.1 | 3.045(1.790-5.237) | 0.2 | 4.398(2.585-7.565) |  |  |  |  |  |  |
| 4.398(2.585 - 7.565) | 10 | 0.2 | 0.1 | 2.984(1.754-5.133) | 0.2 | 4.398(2.585-7.565) | P_C1_ | RR (95%CI) |  |  |  |  |
| 4.398(2.585 - 7.565) | 1 | 0.3 | 0.1 | 4.398(2.585-7.565) | 0.2 | 4.398(2.585-7.565) | 0.3 | 4.398(2.585-7.565) |  |  |  |  |
| 4.398(2.585 - 7.565) | 2 | 0.3 | 0.1 | 3.721(2.187-6.401) | 0.2 | 4.060(2.386-6.983) | 0.3 | 4.398(2.585-7.565) |  |  |  |  |
| 4.398(2.585 - 7.565) | 3 | 0.3 | 0.1 | 3.299(1.939-5.674) | 0.2 | 3.848(2.262-6.619) | 0.3 | 4.398(2.585-7.565) |  |  |  |  |
| 4.398(2.585 - 7.565) | 4 | 0.3 | 0.1 | 3.009(1.769-5.176) | 0.2 | 3.704(2.177-6.371) | 0.3 | 4.398(2.585-7.565) |  |  |  |  |
| 4.398(2.585 - 7.565) | 5 | 0.3 | 0.1 | 2.799(1.645-4.814) | 0.2 | 3.598(2.115-6.190) | 0.3 | 4.398(2.585-7.565) |  |  |  |  |
| 4.398(2.585 - 7.565) | 6 | 0.3 | 0.1 | 2.639(1.551-4.539) | 0.2 | 3.518(2.068-6.052) | 0.3 | 4.398(2.585-7.565) |  |  |  |  |
| 4.398(2.585 - 7.565) | 7 | 0.3 | 0.1 | 2.513(1.477-4.323) | 0.2 | 3.456(2.031-5.944) | 0.3 | 4.398(2.585-7.565) |  |  |  |  |
| 4.398(2.585 - 7.565) | 8 | 0.3 | 0.1 | 2.412(1.418-4.149) | 0.2 | 3.405(2.001-5.857) | 0.3 | 4.398(2.585-7.565) |  |  |  |  |
| 4.398(2.585 - 7.565) | 9 | 0.3 | 0.1 | 2.328(1.369-4.005) | 0.2 | 3.363(1.977-5.785) | 0.3 | 4.398(2.585-7.565) |  |  |  |  |
| 4.398(2.585 - 7.565) | 10 | 0.3 | 0.1 | 2.258(1.327-3.885) | 0.2 | 3.328(1.956-5.725) | 0.3 | 4.398(2.585-7.565) | P_C1_ | RR (95%CI) |  |  |
| 4.398(2.585 - 7.565) | 1 | 0.4 | 0.1 | 4.398(2.585-7.565) | 0.2 | 4.398(2.585-7.565) | 0.3 | 4.398(2.585-7.565) | 0.4 | 4.398(2.585-7.565) |  |  |
| 4.398(2.585 - 7.565) | 2 | 0.4 | 0.1 | 3.456(2.031-5.944) | 0.2 | 3.770(2.216-6.484) | 0.3 | 4.084(2.400-7.025) | 0.4 | 4.398(2.585-7.565) |  |  |
| 4.398(2.585 - 7.565) | 3 | 0.4 | 0.1 | 2.932(1.723-5.043) | 0.2 | 3.421(2.011-5.884) | 0.3 | 3.909(2.298-6.724) | 0.4 | 4.398(2.585-7.565) |  |  |
| 4.398(2.585 - 7.565) | 4 | 0.4 | 0.1 | 2.599(1.528-4.470) | 0.2 | 3.199(1.880-5.502) | 0.3 | 3.798(2.233-6.533) | 0.4 | 4.398(2.585-7.565) |  |  |
| 4.398(2.585 - 7.565) | 5 | 0.4 | 0.1 | 2.368(1.392-4.073) | 0.2 | 3.045(1.790-5.237) | 0.3 | 3.721(2.187-6.401) | 0.4 | 4.398(2.585-7.565) |  |  |
| 4.398(2.585 - 7.565) | 6 | 0.4 | 0.1 | 2.199(1.293-3.783) | 0.2 | 2.932(1.723-5.043) | 0.3 | 3.665(2.154-6.304) | 0.4 | 4.398(2.585-7.565) |  |  |
| 4.398(2.585 - 7.565) | 7 | 0.4 | 0.1 | 2.070(1.216-3.560) | 0.2 | 2.846(1.673-4.895) | 0.3 | 3.622(2.129-6.230) | 0.4 | 4.398(2.585-7.565) |  |  |
| 4.398(2.585 - 7.565) | 8 | 0.4 | 0.1 | 1.968(1.156-3.384) | 0.2 | 2.778(1.633-4.778) | 0.3 | 3.588(2.109-6.171) | 0.4 | 4.398(2.585-7.565) |  |  |
| 4.398(2.585 - 7.565) | 9 | 0.4 | 0.1 | 1.885(1.108-3.242) | 0.2 | 2.723(1.600-4.683) | 0.3 | 3.560(2.093-6.124) | 0.4 | 4.398(2.585-7.565) |  |  |
| 4.398(2.585 - 7.565) | 10 | 0.4 | 0.1 | 1.817(1.068-3.125) | 0.2 | 2.677(1.573-4.605) | 0.3 | 3.538(2.079-6.085) | 0.4 | 4.398(2.585-7.565) | P_C1_ | RR (95%CI) |
| 4.398(2.585 - 7.565) | 1 | 0.5 | 0.1 | 4.398(2.585-7.565) | 0.2 | 4.398(2.585-7.565) | 0.3 | 4.398(2.585-7.565) | 0.4 | 4.398(2.585-7.565) | 0.5 | 4.398(2.585-7.565) |
| 4.398(2.585 - 7.565) | 2 | 0.5 | 0.1 | 3.225(1.896-5.548) | 0.2 | 3.518(2.068-6.052) | 0.3 | 3.812(2.240-6.556) | 0.4 | 4.105(2.413-7.061) | 0.5 | 4.398(2.585-7.565) |
| 4.398(2.585 - 7.565) | 3 | 0.5 | 0.1 | 2.639(1.551-4.539) | 0.2 | 3.079(1.810-5.296) | 0.3 | 3.518(2.068-6.052) | 0.4 | 3.958(2.327-6.809) | 0.5 | 4.398(2.585-7.565) |
| 4.398(2.585 - 7.565) | 4 | 0.5 | 0.1 | 2.287(1.344-3.934) | 0.2 | 2.815(1.654-4.842) | 0.3 | 3.342(1.965-5.749) | 0.4 | 3.870(2.275-6.657) | 0.5 | 4.398(2.585-7.565) |
| 4.398(2.585 - 7.565) | 5 | 0.5 | 0.1 | 2.052(1.206-3.530) | 0.2 | 2.639(1.551-4.539) | 0.3 | 3.225(1.896-5.548) | 0.4 | 3.812(2.240-6.556) | 0.5 | 4.398(2.585-7.565) |
| 4.398(2.585 - 7.565) | 6 | 0.5 | 0.1 | 1.885(1.108-3.242) | 0.2 | 2.513(1.477-4.323) | 0.3 | 3.141(1.846-5.404) | 0.4 | 3.770(2.216-6.484) | 0.5 | 4.398(2.585-7.565) |
| 4.398(2.585 - 7.565) | 7 | 0.5 | 0.1 | 1.759(1.034-3.026) | 0.2 | 2.419(1.422-4.161) | 0.3 | 3.079(1.810-5.296) | 0.4 | 3.738(2.197-6.430) | 0.5 | 4.398(2.585-7.565) |
| 4.398(2.585 - 7.565) | 8 | 0.5 | 0.1 | 1.661(**0.977**-2.858) | 0.2 | 2.346(1.379-4.035) | 0.3 | 3.030(1.781-5.211) | 0.4 | 3.714(2.183-6.388) | 0.5 | 4.398(2.585-7.565) |
| 4.398(2.585 - 7.565) | 9 | 0.5 | 0.1 | 1.583(**0.931**-2.723) | 0.2 | 2.287(1.344-3.934) | 0.3 | 2.991(1.758-5.144) | 0.4 | 3.694(2.171-6.355) | 0.5 | 4.398(2.585-7.565) |
| 4.398(2.585 - 7.565) | 10 | 0.5 | 0.1 | 1.519(**0.893**-2.613) | 0.2 | 2.239(1.316-3.851) | 0.3 | 2.959(1.739-5.089) | 0.4 | 3.678(2.162-6.327) | 0.5 | 4.398(2.585-7.565) |

Table S6 Array Approach Sensitivity Analysis for Unmeasured Confounder That Decreases PE Rate, and Is More Prevalent in the Exposed (Longest Procedures).

The observed relative risk (ARR; approximated by the OR) was set as the lower confidence interval of the pooled odds ratio for the duration of general anesthesia time >3h and PE rate (OR = 4.398; 95% CI = **2.585** - 7.565).

The association between the unmeasured confounder and PE rate(RRCD) was varied from 0.1 to 1.

The prevalence of this confounder was varied from 10% to 50%.

Abbreviations:

ARR = Observed relative risk

RR_CD_ = Association between the unmeasured confounder and PE

P_C0_= Prevalence of the unmeasured confounder in the exposed (duration of general anesthesia time>3h)

P_C1_= Prevalence of the unmeasured confounder in the unexposed (duration of general anesthesia time ≤3h)

RR= Relative risk after adjusting for the unmeasured confounder

$$RR=\frac{\mathrm{ARR}}{\left[ \frac{P_{C1}\left( \mathrm{RR}_{\mathrm{CD}}-1 \right)+1}{P_{C0}\left( \mathrm{RR}_{\mathrm{CD}}-1 \right)+1} \right]}$$

No scenarios would reduce the lower limit of the 95% confidence interval for the pooled odds ratio below 1.00.

| ARR | RR_CD_ | P_C1_ | P_C0_ | RR (95%CI) |  |  |  |  |  |  |  |  |
| --- | --- | --- | --- | --- | --- | --- | --- | --- | --- | --- | --- | --- |
| 4.398(2.585 - 7.565) | 0.1 | 0.1 | 0.1 | 4.398(2.585-7.565) |  |  |  |  |  |  |  |  |
| 4.398(2.585 - 7.565) | 0.2 | 0.1 | 0.1 | 4.398(2.585-7.565) |  |  |  |  |  |  |  |  |
| 4.398(2.585 - 7.565) | 0.3 | 0.1 | 0.1 | 4.398(2.585-7.565) |  |  |  |  |  |  |  |  |
| 4.398(2.585 - 7.565) | 0.4 | 0.1 | 0.1 | 4.398(2.585-7.565) |  |  |  |  |  |  |  |  |
| 4.398(2.585 - 7.565) | 0.5 | 0.1 | 0.1 | 4.398(2.585-7.565) |  |  |  |  |  |  |  |  |
| 4.398(2.585 - 7.565) | 0.6 | 0.1 | 0.1 | 4.398(2.585-7.565) |  |  |  |  |  |  |  |  |
| 4.398(2.585 - 7.565) | 0.7 | 0.1 | 0.1 | 4.398(2.585-7.565) |  |  |  |  |  |  |  |  |
| 4.398(2.585 - 7.565) | 0.8 | 0.1 | 0.1 | 4.398(2.585-7.565) |  |  |  |  |  |  |  |  |
| 4.398(2.585 - 7.565) | 0.9 | 0.1 | 0.1 | 4.398(2.585-7.565) |  |  |  |  |  |  |  |  |
| 4.398(2.585 - 7.565) | 1 | 0.1 | 0.1 | 4.398(2.585-7.565) | P_C0_ | RR (95%CI) |  |  |  |  |  |  |
| 4.398(2.585 - 7.565) | 0.1 | 0.2 | 0.1 | 4.881(2.869-8.395) | 0.2 | 4.398(2.585-7.565) |  |  |  |  |  |  |
| 4.398(2.585 - 7.565) | 0.2 | 0.2 | 0.1 | 4.817(2.831-8.285) | 0.2 | 4.398(2.585-7.565) |  |  |  |  |  |  |
| 4.398(2.585 - 7.565) | 0.3 | 0.2 | 0.1 | 4.756(2.795-8.181) | 0.2 | 4.398(2.585-7.565) |  |  |  |  |  |  |
| 4.398(2.585 - 7.565) | 0.4 | 0.2 | 0.1 | 4.698(2.761-8.081) | 0.2 | 4.398(2.585-7.565) |  |  |  |  |  |  |
| 4.398(2.585 - 7.565) | 0.5 | 0.2 | 0.1 | 4.642(2.729-7.985) | 0.2 | 4.398(2.585-7.565) |  |  |  |  |  |  |
| 4.398(2.585 - 7.565) | 0.6 | 0.2 | 0.1 | 4.589(2.697-7.894) | 0.2 | 4.398(2.585-7.565) |  |  |  |  |  |  |
| 4.398(2.585 - 7.565) | 0.7 | 0.2 | 0.1 | 4.538(2.668-7.806) | 0.2 | 4.398(2.585-7.565) |  |  |  |  |  |  |
| 4.398(2.585 - 7.565) | 0.8 | 0.2 | 0.1 | 4.490(2.639-7.723) | 0.2 | 4.398(2.585-7.565) |  |  |  |  |  |  |
| 4.398(2.585 - 7.565) | 0.9 | 0.2 | 0.1 | 4.443(2.611-7.642) | 0.2 | 4.398(2.585-7.565) |  |  |  |  |  |  |
| 4.398(2.585 - 7.565) | 1 | 0.2 | 0.1 | 4.398(2.585-7.565) | 0.2 | 4.398(2.585-7.565) | P_C0_ | RR (95%CI) |  |  |  |  |
| 4.398(2.585 - 7.565) | 0.1 | 0.3 | 0.1 | 5.482(3.222-9.430) | 0.2 | 4.940(2.904-8.498) | 0.3 | 4.398(2.585-7.565) |  |  |  |  |
| 4.398(2.585 - 7.565) | 0.2 | 0.3 | 0.1 | 5.324(3.129-9.158) | 0.2 | 4.861(2.857-8.361) | 0.3 | 4.398(2.585-7.565) |  |  |  |  |
| 4.398(2.585 - 7.565) | 0.3 | 0.3 | 0.1 | 5.177(3.043-8.906) | 0.2 | 4.788(2.814-8.235) | 0.3 | 4.398(2.585-7.565) |  |  |  |  |
| 4.398(2.585 - 7.565) | 0.4 | 0.3 | 0.1 | 5.042(2.963-8.672) | 0.2 | 4.720(2.774-8.119) | 0.3 | 4.398(2.585-7.565) |  |  |  |  |
| 4.398(2.585 - 7.565) | 0.5 | 0.3 | 0.1 | 4.915(2.889-8.455) | 0.2 | 4.657(2.737-8.010) | 0.3 | 4.398(2.585-7.565) |  |  |  |  |
| 4.398(2.585 - 7.565) | 0.6 | 0.3 | 0.1 | 4.798(2.820-8.253) | 0.2 | 4.598(2.703-7.909) | 0.3 | 4.398(2.585-7.565) |  |  |  |  |
| 4.398(2.585 - 7.565) | 0.7 | 0.3 | 0.1 | 4.688(2.755-8.064) | 0.2 | 4.543(2.670-7.814) | 0.3 | 4.398(2.585-7.565) |  |  |  |  |
| 4.398(2.585 - 7.565) | 0.8 | 0.3 | 0.1 | 4.585(2.695-7.887) | 0.2 | 4.492(2.640-7.726) | 0.3 | 4.398(2.585-7.565) |  |  |  |  |
| 4.398(2.585 - 7.565) | 0.9 | 0.3 | 0.1 | 4.489(2.638-7.721) | 0.2 | 4.443(2.612-7.643) | 0.3 | 4.398(2.585-7.565) |  |  |  |  |
| 4.398(2.585 - 7.565) | 1 | 0.3 | 0.1 | 4.398(2.585-7.565) | 0.2 | 4.398(2.585-7.565) | 0.3 | 4.398(2.585-7.565) | P_C0_ | RR (95%CI) |  |  |
| 4.398(2.585 - 7.565) | 0.1 | 0.4 | 0.1 | 6.253(3.676-10.756) | 0.2 | 5.635(3.312-9.693) | 0.3 | 5.016(2.949-8.629) | 0.4 | 4.398(2.585-7.565) |  |  |
| 4.398(2.585 - 7.565) | 0.2 | 0.4 | 0.1 | 5.950(3.497-10.235) | 0.2 | 5.433(3.193-9.345) | 0.3 | 4.915(2.889-8.455) | 0.4 | 4.398(2.585-7.565) |  |  |
| 4.398(2.585 - 7.565) | 0.3 | 0.4 | 0.1 | 5.681(3.339-9.771) | 0.2 | 5.253(3.088-9.036) | 0.3 | 4.826(2.836-8.300) | 0.4 | 4.398(2.585-7.565) |  |  |
| 4.398(2.585 - 7.565) | 0.4 | 0.4 | 0.1 | 5.440(3.197-9.357) | 0.2 | 5.092(2.993-8.759) | 0.3 | 4.745(2.789-8.162) | 0.4 | 4.398(2.585-7.565) |  |  |
| 4.398(2.585 - 7.565) | 0.5 | 0.4 | 0.1 | 5.223(3.070-8.983) | 0.2 | 4.948(2.908-8.511) | 0.3 | 4.673(2.747-8.038) | 0.4 | 4.398(2.585-7.565) |  |  |
| 4.398(2.585 - 7.565) | 0.6 | 0.4 | 0.1 | 5.026(2.954-8.646) | 0.2 | 4.817(2.831-8.285) | 0.3 | 4.607(2.708-7.925) | 0.4 | 4.398(2.585-7.565) |  |  |
| 4.398(2.585 - 7.565) | 0.7 | 0.4 | 0.1 | 4.848(2.849-8.339) | 0.2 | 4.698(2.761-8.081) | 0.3 | 4.548(2.673-7.823) | 0.4 | 4.398(2.585-7.565) |  |  |
| 4.398(2.585 - 7.565) | 0.8 | 0.4 | 0.1 | 4.685(2.754-8.058) | 0.2 | 4.589(2.697-7.894) | 0.3 | 4.494(2.641-7.729) | 0.4 | 4.398(2.585-7.565) |  |  |
| 4.398(2.585 - 7.565) | 0.9 | 0.4 | 0.1 | 4.535(2.666-7.801) | 0.2 | 4.490(2.639-7.723) | 0.3 | 4.444(2.612-7.644) | 0.4 | 4.398(2.585-7.565) |  |  |
| 4.398(2.585 - 7.565) | 1 | 0.4 | 0.1 | 4.398(2.585-7.565) | 0.2 | 4.398(2.585-7.565) | 0.3 | 4.398(2.585-7.565) | 0.4 | 4.398(2.585-7.565) | P_C0_ | RR (95%CI) |
| 4.398(2.585 - 7.565) | 0.1 | 0.5 | 0.1 | 7.277(4.277-12.517) | 0.2 | 6.557(3.854-11.279) | 0.3 | 5.837(3.431-10.041) | 0.4 | 5.118(3.008-8.803) | 0.5 | 4.398(2.585-7.565) |
| 4.398(2.585 - 7.565) | 0.2 | 0.5 | 0.1 | 6.744(3.964-11.600) | 0.2 | 6.157(3.619-10.591) | 0.3 | 5.571(3.274-9.582) | 0.4 | 4.984(2.930-8.574) | 0.5 | 4.398(2.585-7.565) |
| 4.398(2.585 - 7.565) | 0.3 | 0.5 | 0.1 | 6.293(3.699-10.824) | 0.2 | 5.819(3.420-10.009) | 0.3 | 5.345(3.142-9.194) | 0.4 | 4.872(2.863-8.380) | 0.5 | 4.398(2.585-7.565) |
| 4.398(2.585 - 7.565) | 0.4 | 0.5 | 0.1 | 5.906(3.471-10.159) | 0.2 | 5.529(3.250-9.510) | 0.3 | 5.152(3.028-8.862) | 0.4 | 4.775(2.807-8.213) | 0.5 | 4.398(2.585-7.565) |
| 4.398(2.585 - 7.565) | 0.5 | 0.5 | 0.1 | 5.571(3.274-9.582) | 0.2 | 5.278(3.102-9.078) | 0.3 | 4.984(2.930-8.574) | 0.4 | 4.691(2.757-8.069) | 0.5 | 4.398(2.585-7.565) |
| 4.398(2.585 - 7.565) | 0.6 | 0.5 | 0.1 | 5.278(3.102-9.078) | 0.2 | 5.058(2.973-8.700) | 0.3 | 4.838(2.844-8.322) | 0.4 | 4.618(2.714-7.943) | 0.5 | 4.398(2.585-7.565) |
| 4.398(2.585 - 7.565) | 0.7 | 0.5 | 0.1 | 5.019(2.950-8.633) | 0.2 | 4.864(2.859-8.366) | 0.3 | 4.708(2.767-8.099) | 0.4 | 4.553(2.676-7.832) | 0.5 | 4.398(2.585-7.565) |
| 4.398(2.585 - 7.565) | 0.8 | 0.5 | 0.1 | 4.789(2.815-8.237) | 0.2 | 4.691(2.757-8.069) | 0.3 | 4.593(2.700-7.901) | 0.4 | 4.496(2.642-7.733) | 0.5 | 4.398(2.585-7.565) |
| 4.398(2.585 - 7.565) | 0.9 | 0.5 | 0.1 | 4.583(2.694-7.884) | 0.2 | 4.537(2.667-7.804) | 0.3 | 4.491(2.639-7.724) | 0.4 | 4.444(2.612-7.645) | 0.5 | 4.398(2.585-7.565) |
| 4.398(2.585 - 7.565) | 1 | 0.5 | 0.1 | 4.398(2.585-7.565) | 0.2 | 4.398(2.585-7.565) | 0.3 | 4.398(2.585-7.565) | 0.4 | 4.398(2.585-7.565) | 0.5 | 4.398(2.585-7.565) |

Reference

1. Kyriacou, D.N. and R.J. Lewis, *Confounding by Indication in Clinical Research.* Jama, 2016. **316**(17): p. 1818-1819.

2. Thomas, L.E., F. Li, and M.J. Pencina, *Overlap Weighting: A Propensity Score Method That Mimics Attributes of a Randomized Clinical Trial.* Jama, 2020. **323**(23): p. 2417-2418.

3. Li, F., L.E. Thomas, and F. Li, *Addressing Extreme Propensity Scores via the Overlap Weights.* Am J Epidemiol, 2019. **188**(1): p. 250-257.

4. Schneeweiss, S., *Sensitivity analysis and external adjustment for unmeasured confounders in epidemiologic database studies of therapeutics.* Pharmacoepidemiol Drug Saf, 2006. **15**(5): p. 291-303.
